# A Study of Older Adults’ Mental Health across 33 Countries during the COVID-19 Pandemic

**DOI:** 10.3390/ijerph18105090

**Published:** 2021-05-11

**Authors:** Carmen M. Tyler, Grace B. McKee, Elisabet Alzueta, Paul B. Perrin, Kristine Kingsley, Fiona C. Baker, Juan Carlos Arango-Lasprilla

**Affiliations:** 1 Department of Psychology, Virginia Commonwealth University, Richmond, VA 23284, USA; tylercm2@vcu.edu (C.M.T.); or grace.mckee@va.gov (G.B.M.); pperrin@vcu.edu (P.B.P.); 2 Advanced Fellowship Program in Mental Illness Research and Treatment, Mid-Atlantic Mental Illness Research Education and Clinical Center (MIRECC), Central Virginia VA Health Care System, Richmond, VA 23249, USA; 3 Center for Health Sciences, SRI International, Menlo Park, CA 94025, USA; elisabet.alzueta@sri.com (E.A.); fiona.baker@sri.com (F.C.B.); 4 Department of Biological and Health Psychology, Autonomous University of Madrid, 28049 Madrid, Spain; 5 Institute of Cognitive and Emotional Wellness, Westchester, NY 10801, USA; cognitiveandemotionalwellness@gmail.com; 6 Department of Physiology, University of the Witwatersrand, Johannesburg 2050, South Africa; 7 BioCruces Health Research Institute, 48903 Barakaldo, Spain; 8 IKERBASQUE, Basque Foundation for Science, 48009 Bilbao, Spain; 9 Department of Cell Biology and Histology, University of the Basque Country UPV/EHU, Leioa, 48940 Vizcaya, Spain

**Keywords:** older adults, COVID-19, coronavirus, pandemic, depression, anxiety, mental health, international

## Abstract

Despite older adults’ extremely high vulnerability to COVID-19 complications and death, few studies have examined how personal characteristics and the COVID-19 pandemic have impacted the mental health of older adults at the global level. The purpose of this study was to examine the relationships among demographics, COVID-19 life impacts, and depression and anxiety in adults aged 60 and older from 33 countries. A sample of 823 older adults aged 60–94 and residing in 33 countries completed a 10-min online survey following recruitment from mailing lists and social media. Being separated from and having conflicts with loved ones predicted both anxiety and depression, as did residing in a country with higher income. Getting medical treatment for severe symptoms of COVID-19 and having decreased work responsibilities predicted depression, but adjustment to working from home and younger age predicted both depression and anxiety. Participants from Europe and Central Asia reported higher depression than those from all other regions and higher anxiety than those from Latin America and the Caribbean. The COVID-19 pandemic has had serious deleterious effects on the mental health of older adults worldwide. The current findings have direct implications for mental health services that may be delivered to older adults to help facilitate healthy psychological adjustment.

## 1. Introduction

In March 2020, the World Health Organization (WHO) declared that the rapid worldwide spread of the new type of coronavirus (popularly known as COVID-19), first diagnosed only a few months prior in China’s Hubei Province, was far-reaching enough to be classified as a pandemic [1]. WHO then issued recommendations for physical distancing to minimize the risk of interpersonal transmission of the virus [2], and governments imposed varying degrees of “stay at home” or quarantine requirements for their citizens [3]. In addition to heightened fears associated with the possibility of contracting a disease which may or may not respond well to treatment, studies from multiple international teams showed that government- and self-imposed restrictions on movement and social interactions related to the pandemic took their toll with reports of increased anxiety, depression, and substance use, and lower overall mental health for the general population [3,4,5,6,7,8,9,10]. Legitimate reasons for why authorities and systems enacted these measures do not negate the psychosocial consequences of enforced isolation. In people who were being treated for mental health conditions prior to the pandemic, fear, depression, and anxiety levels increased to the point that they were sometimes surpassing the original presenting complaints [11]. Efforts to control the spread of COVID-19 by limiting social contact diminished opportunities to employ the fundamental coping strategy of seeking support from others [12] when distressed, as people were discouraged from gathering with anyone outside their household. Constant news of infection rates and death tolls bombarded the public, and this may also have adversely affected mental wellbeing [13,14,15]. 

Distress regarding COVID-19 may be amplified in older adults [16], as they have been among the most vulnerable with regard to morbidity and mortality from the virus, with 8 out of 10 deaths in those 65 and older (by May 2020, 10% of northern Italy’s older population had died; [17]). Additionally, hospital readmission rates for those with chronic illnesses often seen in older adulthood (e.g., diabetes, heart failure, chronic kidney disease, and respiratory illnesses; [18]) are higher, and medications used to treat these illnesses (i.e., angiotensin-converting enzyme inhibitors and angiotensin II receptor blockers) may actually enhance the mechanism of infection [19]. 

Economic factors may also play a part in stressors related to the pandemic, as one in five older adults in the U.S. resides in areas where economic insecurity and high viral rates intersect [20]. Nearly half the adults surveyed in a study from Cyprus (*n* = 1642) reported they were very concerned about their finances, and two-thirds said that they had large changes in their quality of life [21]. Economic effects may be especially concerning when considering the macroeconomic impact of the virus, with shortages of available goods leading to higher prices [22] and the possibility that older adults may have had to use their retirement savings and having to incur the age-related risks of contracting the virus if they try to reenter the job market [23]. 

Additionally, older adults may feel the impact of required social isolation to an even greater degree than other age groups [24], as more than one-third of people aged 75 or older in a Finnish study (*n* = 6786) reported being lonely irrespective of the current pandemic [25], and a study of social isolation from the National Health and Aging Trends Study estimated that in 2011 about 24% of adults 65 and older were socially isolated [26]. Social isolation can have physical and emotional impacts [27,28] such as increased stress and fear [29] in the short term and in the long term [30,31]. In a setting of mandated prolonged confinement, older adults may experience depressive symptoms, loneliness, pessimism, deteriorations in cognition, and disruption in sleeping patterns [32], consistent with known psychological reactions in a pandemic such as stress, anxiety, loneliness, and agitation [33]. Depression and loneliness increased in older adults during the early months of the pandemic when compared to pre-pandemic levels; for those with closer relationships, greater loneliness predicted deeper depression, and for those who perceived their social relationships to be more distant, depression levels were still higher than they had been before the pandemic [34].

Emerging literature is documenting the COVID-19 paradox of physical protective measures instigating and exacerbating mental health problems [8]. However, few studies have examined the interplay among personal characteristics, COVID-19-impacts on one’s life, and mental health in older adults, despite older adults’ extremely high vulnerability to COVID-19 complications in the global population. Such an examination is imperative in informing the development and implementation of effective interventions to safeguard their mental health. Therefore, the purpose of this study was to examine the relationships among demographics, COVID-19 life impacts, and depression and anxiety in older adults internationally. Specifically, demographic variables, personal COVID-19 exposure, quarantine level, and pandemic-related life changes were explored as predictors of depression and anxiety in adults aged 60 and older from 33 countries. 

## 2. Materials and Methods

### 2.1. Participants

**Figure 1:**
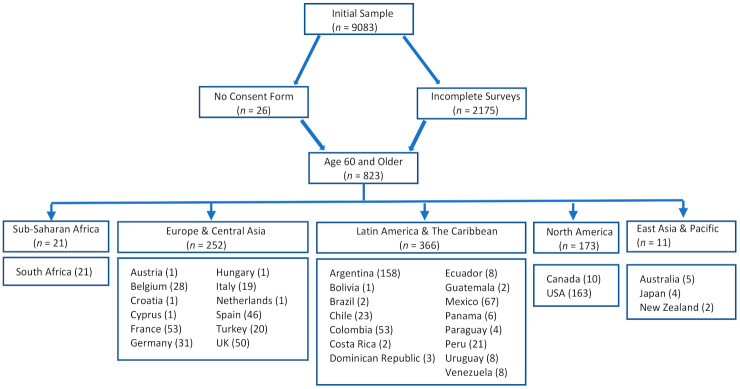
Flowchart of participant inclusion/exclusion process.

The current study’s participants comprised the subset of all of those who were aged 60 or older in a larger international study of COVID-19 impacts on mental health [4]. This subsample of 823 participants ranging in age from 60–94 (Mean age: 66.13 ± 5.50 years) and residing in 33 countries (26 countries from the larger study had no participants aged 60 or older and were therefore excluded from the current study) were retained for the current analyses (see Figure 1 for inclusion/exclusion process). Participant demographic characteristics appear in Table 1. 

### 2.2. Measures

#### 2.2.1. Quarantine Level

Participants were asked to select the level of social restrictions they were following in the previous 2 weeks due to the COVID-19 pandemic. Four different levels of restrictions were specified: Level 0: “no specific restrictions”; Level 1: “mild restrictions (e.g., not gathering with 10 or more people, not traveling outside my city or state)”; Level 2: “moderate restrictions (e.g., not leaving home except for working, care of another family member, exercise, or getting fresh air)”; Level 3: “severe restrictions (e.g., not leaving home at all, or only leaving to buy food or medicine).” 

#### 2.2.2. Epidemic–Pandemic Impacts Inventory (EPII)

The EPII [35] was created by the University of Connecticut School of Medicine as a measure of the impacts of epidemics and pandemics across personal and social life domains. For the purposes of this study, we included only those EPII items related to exposure to COVID-19 (seven “yes/no” questions) and its impacts on life (13 “yes/no” questions) (α = 0.66). The original instructions for the EPII were adapted slightly to specifically relate to the COVID-19 pandemic: “Since the coronavirus pandemic began, what has changed for you?”

#### 2.2.3. Depression, Anxiety and Stress Scale-21 (DASS-21)

The DASS-21 [36] is a 21-item measure, which includes subscales for stress, anxiety, and depression. For this study, we used only the depression subscale, which is a measure of hopelessness, low self-esteem, and low positive affect. Each of its seven items is rated on a 4-point Likert scale. Total summed score indicates the presence and severity of depression symptoms, with higher scores indicating greater depression: 0–4 indicating normal range, 5–6 indicating mild depression, 7–10 indicating moderate depression, 11–13 indicating severe depression, and 14 and greater indicating extremely severe depression. The DASS-21 has been shown to have good psychometric properties and good validity for older populations [37]. 

#### 2.2.4. Generalized Anxiety Disorder-7 (GAD-7)

The GAD-7 [38] measures anxiety and worry during the past 2 weeks using a 4-point Likert scale for its seven items. Summed total scores may range from 0 to 21, where higher scores indicate higher levels of anxiety symptoms (0–4 = minimal; 5–9 = mild; 10–14 = moderate; 15–21 = severe). This measure has shown strong reliability, construct validity, internal consistency, and convergent validity [39,40].

### 2.3. Procedure

The current study is a secondary analysis of cross-sectional survey data collected for a larger study [4]. In the original study, an online survey was created using a combination of both standardized and adapted measures for the general population. The original survey was translated from English into five different additional languages (Italian, French, Spanish, Turkish, and German) by native speakers. For the standardized measures, the validated version for each language and culture was used. The online survey was hosted on SurveyMonkey and distributed via a snowball sampling method using mailing lists and social media (i.e., Facebook, Instagram, Twitter, and WhatsApp). Data were collected from 19 April 2020 to 3 May 2020. Participants were apprised of the study aims before giving their informed consent. Participation was anonymous and voluntary—participants did not receive any monetary compensation for their participation. This study was conducted in compliance with the declaration of Helsinki and approved by the Autonomous University of Madrid Ethical Committee (CEI-106-206).

### 2.4. Data Analysis

All analyses were conducted using IBM SPSS 26. Participants’ home countries were classified by World Bank gross national income (GNI) per capita estimates in U.S. dollars (low: GNI ≤ $1035; lower-middle: GNI = $1035–$4045; upper-middle: GNI = $4046–$12,535; and high: GNI ≥ $12,536) and by World Bank designated geographical regions (i.e., East Asia and Pacific, Europe and Central Asia, Latin America and the Caribbean, North America, Sub-Saharan Africa, and South Asia [41]). Table 2 shows a bivariate correlation matrix of the associations among depression, anxiety, and demographic characteristics. In order to test the statistical effects of demographic characteristics, COVID-19-related variables, and effects of the pandemic on people’s lives as predictors of depression and anxiety, two hierarchical stepwise multiple linear regressions were computed with depression and anxiety as the respective outcome variables. In both regressions, Step 1 included demographic variables (i.e., identifying as a man vs. woman or non-binary/trans, age, country income classification, partnered vs. not partnered, active vs. inactive work status, and dependent <18 years in the home vs. none). Variables reflecting participants’ own exposure to COVID-19 were included in Step 2; zero participants in the current sample positively endorsed the item “Hospital stay due to this disease,” so it was subsequently excluded from analyses. Local level of quarantine was included in Step 3, and Step 4 included pandemic-related effects on participants’ lives. Second, depression and anxiety scores were compared by global region using two separate analyses of covariance (ANCOVAs). The demographic variables included in Step 1 of the previous hierarchical regressions were included in these ANCOVAs as covariates. 

## 3. Results

### 3.1. Correlation Matrix 

In the correlation matrix (Table 2), identifying as a woman or trans/non-binary, not being partnered, and living in a higher income country were associated with higher depression. Depression was not associated with age, employment status, or having a dependent child under 18 years of age in the home. Identifying as a woman or trans/non-binary, younger age, and living in a higher income country were associated with higher anxiety. Anxiety was not associated with being partnered vs. not partnered, employment status, or having a dependent child under 18 years of age in the home. 

### 3.2. Predictors of Depression during the COVID-19 Pandemic

In the hierarchical linear regression predicting depression, Step 1 was statistically significant, *F*(6, 816) = 12.27, *R*^2^ = 0.083, *p* < 0.001. Consistent with the correlation matrix, depression was significantly and uniquely predicted by identifying as a woman or trans/non-binary and living in a higher income country. However, inconsistent with the bivariate correlations, higher depression was also associated with younger age and with not being partnered. With the addition of predictors reflecting COVID-19 exposure in Step 2, the overall model was still statistically significant, *F*(13, 809) = 6.69, *R*^2^ = 0.097, *p* < 0.001. Within this step, having received medical treatment due to severe symptoms of the disease was associated with higher depression, *p* = 0.003. Stricter quarantine level (Step 3) did not significantly predict depression, *p* = 0.199, but the overall model remained significant, *F*(14, 808) = 6.33, *R*^2^ = 0.099, *p* < 0.001. 

After the Step 4 addition of pandemic-related effects on participants’ lives, the overall model was still significant, *F*(27, 795) = 6.20, *R*^2^ = 0.174, *p* < 0.001, explaining 17.4% of the variance in depression. Within this step, significant predictors of higher depression included lower workload or work responsibilities, having a hard time transitioning to working from home, increases in verbal arguments or conflict with other adults in the home, and being separated from family or close friends. Notably, country income classification and having increased verbal conflicts with other adults in the home emerged as the two strongest predictors of depression out of all predictor variables in the model at Step 4. Two variables that significantly predicted depression in previous steps (gender and relationship status) were no longer significant at the *p* < 0.05 level after the addition of Step 4 predictors. Table 3 presents all statistical effects on depression from Step 4, as well as percentages and means of the predictors endorsed. 

### 3.3. Predictors of Anxiety during the COVID-19 Pandemic

In the hierarchical linear regression predicting anxiety, Step 1 was statistically significant, *F*(6, 816) = 7.54, *R*^2^ = 0.052, *p* < 0.001. Consistent with the correlation matrix, anxiety was significantly predicted by identifying as a woman or trans/non-binary, younger age, and higher country income classification. None of the COVID-19-related exposure predictors included in Step 2 significantly predicted anxiety, although the overall model was still statistically significant, *F*(13, 809) = 4.51, *R*^2^ = 0.068, *p* < 0.001. Similarly, stricter quarantine level (Step 3) did not predict anxiety, though the overall model remained significant, *F*(14, 808) = 4.31, *R*^2^ = 0.070, *p* < 0.001.

After pandemic-related effects on participants’ lives were added in Step 4, the overall model was still significant, *F*(27, 795) = 5.93, *R*^2^ = 0.168, *p* < 0.001. Within this step, having a hard time transitioning to working from home, having increases in verbal arguments or conflict with other adults in the home, and being separated from family or close friends were all significantly associated with higher anxiety. Again, country income classification and increased conflicts with other adults in the home emerged as the two strongest predictors out of all predictor variables at Step 4. Gender, which significantly predicted anxiety in previous steps, was no longer significant at *p* < 0.05 after the addition of Step 4 predictors. Table 3 presents all statistical effects on anxiety from Step 4.

### 3.4. Differences in Depression across Global Regions during the Pandemic

**Figure 2:**
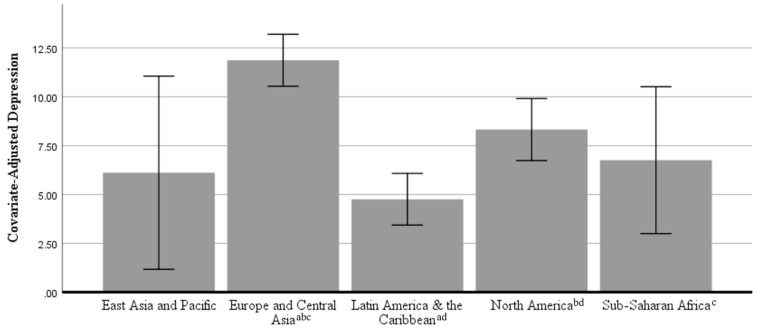
Covariate-Adjusted Depression Scores (Mean with 95% Confidence Intervals) by Global Region. Note: Means were adjusted for the following covariates: gender, age, country income classification, marital status, work status, and having dependents in the home. Regions sharing the same superscript were significantly different at *p* < 0.05 after Bonferroni corrections.

In the ANCOVA predicting depression, there was a statistically significant effect of global region, *F*(4, 812) = 12.483, partial eta^2^ = 0.058, *p* < 0.001. Figure 2 shows the covariate-adjusted estimated marginal means for depression with error bars representing 95% confidence intervals. Bonferroni-corrected post-hoc pairwise comparisons showed that participants from Europe and Central Asia (*M* = 11.87, *SD* = 10.40) reported significantly higher depression than participants from Latin America and the Caribbean (*M* = 4.96, *SD* = 6.38), *p* < 0.001; participants from North America (*M* = 8.05, *SD* = 8.40), *p* < 0.001; and participants from Sub-Saharan Africa (*M* = 5.81, *SD* = 6.66), *p* = 0.012. Participants from North America reported significantly higher depression than did participants from Latin America and the Caribbean, *p* = 0.001. No significant differences were observed at the *p* < 0.05 level among participants from East Asia and the Pacific (*M* = 5.64, *SD* = 4.46) and the other global regions. Please see Table 4 for a breakdown of depression levels by country.

### 3.5. Differences in Anxiety across Global Regions during the Pandemic

**Figure 3:**
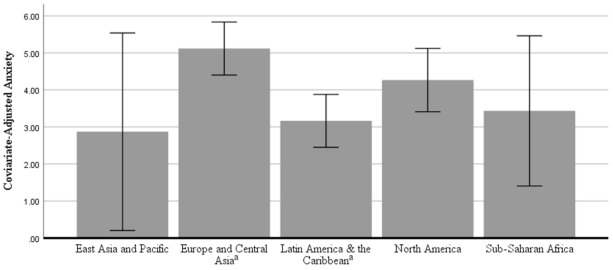
Covariate-adjusted anxiety scores (mean with 95% confidence intervals) by global region. Note: Means were adjusted for the following covariates: gender, age, country income classification, marital status, work status, and having dependents in the home. Regions sharing the same superscript were significantly different at *p* < 0.05 after Bonferroni corrections.

In the ANCOVA predicting anxiety, there was a significant effect of global region, *F*(4, 812) = 3.269, partial eta^2^ = 0.016, *p* = 0.011. Figure 3 shows the covariate-adjusted estimated marginal means for anxiety with error bars representing 95% confidence intervals. Bonferroni-corrected post hoc pairwise comparisons showed that participants from Latin America and the Caribbean (*M* = 3.30, *SD* = 3.87) reported significantly lower anxiety than participants from Europe and Central Asia (*M* = 5.10, *SD* = 5.09). No other significant differences emerged at the *p* < 0.05 level among participants from East Asia and the Pacific (*M* = 2.45, *SD* = 2.42), North America (*M* = 4.13, *SD* = 4.79), or Sub-Saharan Africa (*M* = 2.67, *SD* = 4.22). Please see Table 4 for a breakdown of anxiety levels by country.

## 4. Discussion

This multinational study was undertaken to explore personal and environmental factors potentially affecting the depression and anxiety levels of older adults during the COVID-19 pandemic, as they represent the most vulnerable group overall for pandemic-related death and prolonged illness. The most notable findings were that being separated from family and close friends predicted both anxiety and depression but having arguments or conflicts with other adults in the home was the largest predictor of anxiety and the second largest predictor of depression. Higher country income was the biggest predictor of depression and the second largest predictor of anxiety. Receiving medical treatment for severe symptoms of COVID-19 predicted depression. Having a hard time adjusting to working from home predicted depression and anxiety, and having increased work responsibilities inversely predicted depression. Younger age predicted more depression and anxiety. Global differences in depression and anxiety emerged such that participants from Europe and Central Asia had higher depression levels than those from Latin America and the Caribbean, North America, and Sub-Saharan Africa. Participants from North America had higher depression than did those from Latin America and the Caribbean, who had lower anxiety than participants from Europe and Central Asia.

This study found that higher levels of depression and anxiety in older adults during the pandemic were predicted by identification as a woman or as trans/non-binary, which would be consistent with depression findings for the general population of older adults even in non-pandemic situations [42,43]. However, it should be noted that due to the very small number of individuals who identified as trans/non-binary in this study, these results were most likely due to gender differences in depression between men and women. Furthermore, when pandemic-related life impact variables were added, gender differences no longer predicted either anxiety or depression, pointing to the overarching influence of the pandemic-related changes people have been experiencing, or differential impact on women relative to men. Similar to findings in the Perrin et al. study [11], where fear, anxiety, and depression regarding the pandemic overshadowed patients’ original mental health concerns, risk factors typically associated with depression and anxiety may lose precedence in light of the changes the pandemic has brought. 

Living in a country with a higher income was correlated with both depression and anxiety and emerged as the variable that most strongly predicted depression and was second largest in predicting anxiety when all other variables had been added to the model. This finding almost perfectly parallels those from the ANCOVAs comparing the mental health of participants from different global regions. In terms of the mean, participants from Europe and Central Asia, followed by those from North America, had the highest depression and anxiety levels, though only some pairwise comparisons to other regions were statistically significant. This finding is consistent with previous studies showing residents of higher-income countries reporting higher lifetime depression rates [42]. One explanation is the theory that the greater income inequality within countries with higher average incomes is responsible for decreased wellbeing in the people in those countries [44]. This income inequity may be even more pronounced for older adults who are often on fixed incomes which are substantially lower than what they previously earned and lower than those of younger people living around them. However, this finding may also reflect the pattern of the pandemic’s spread globally. At the time of data collection, the regions with the highest contagion rates were Europe and Central Asia and North America [45], traditionally the areas comprising countries with comparatively higher average incomes. It is reasonable, therefore, to expect depression and anxiety rates to be higher in countries more deeply affected by the virus at that time. 

Our sample was relatively young in terms of being older adults, with a mean age of 66, so it is not surprising that 41% of the sample was still employed. As COVID-19 rampaged across the world, the number of available workers was reduced in several ways: personal contraction of the virus, caring for family members who had contracted the virus, childcare for children mandated to stay at home from school or daycare, need to self-quarantine because of exposure to an infected person, or designation as a non-essential employee. Many employers have required their employees to work virtually, which not only has necessitated adapting to and managing a home work environment, but may also have entailed learning new technological skills and purchasing supplemental equipment and supplies. Additional family responsibilities and changes in the usual home environment in conjunction with the need to quickly become proficient at adapting to a virtual work domain may have contributed to difficulty in making the change to working from home; this could explain the current findings that older adults who experienced difficulty with the transition had more depression and anxiety. Nonetheless, an increase in workload responsibilities in the current study was actually associated with *decreased* depression, perhaps reflecting meaningful or productive activity helping older adults maintain a sense of normalcy and continuation in employment identity roles important to their psychological wellbeing [46,47]. Being unable to work because of pandemic-induced changes may affect mental health in ways that parallel those associated with retirement, especially involuntary retirement, which has been shown to negatively impact the mental health of older adults [48,49]. It is also possible that higher rates of depression for those who had received treatment for COVID-19 symptoms could be tied to economic impacts of experiencing the illness—being unable to work or having limited medical resources or public transportation available. The current study thus added to our understanding of the role of employment in older adulthood by (1) confirming that many older adults are working past traditional ages of retirement; (2) suggesting that remaining in the workforce may constitute a meaningful activity for older adults, thus contributing to mental wellbeing; and (3) illustrating the possible economic importance of continued employment for older adults and the implications for mental health. 

Additionally, the current study found that being unpartnered was a predictor of higher depression levels, consistent with findings in non-pandemic times [42] across the globe [50]. Although this study did not find that stricter quarantine policies predicted depression or anxiety, it is possible that the quarantine-induced isolation was felt more deeply in those without partners. In older adults who were unpartnered, receiving treatment for severe symptoms of COVID-19 may also have contributed to increased social isolation and risk of developing depression because of the danger of transmission to potential caregivers who lived apart from the older adult. In the pandemic milieu, older adults without partners may have more difficulty connecting with others in their social network and thus may be more vulnerable for developing depression and anxiety. 

Adults tend to condense their social networks as they age, retaining those with whom they feel the closest and pruning other relationships; those who comprise this smaller social circle then play a more important role in the older adult’s network, and they tend to be those who bring mostly positive emotional experiences [51]. This smaller but closer social network could account for findings that in the initial months of the pandemic older adults perceived higher social support than did middle-aged and young adults [52]. Emotional expression by the people who make up the inner circle predicts emotional experience of the older adult, especially when it is negative [51], and it is therefore understandable that increased verbal arguments or conflicts with other adults in the home would be one of the strongest predictors of depression and anxiety in older adults. Likewise, being separated from family or close friends who have become part of that tighter social net could also be keenly felt, especially in the prolonged separations brought about by COVID-19, and this predicted both anxiety and depression in the current study. These findings were congruent with an international study conducted in early Spring 2020, which found that a protective factor for mental health during the pandemic included the ability to engage with family and friends in problem sharing [53]. The perception of social support is especially important in the development of depression in frail older adults [54]. The current study lends support to previous work on the importance of social support and emotional development in older adults (e.g., socioemotional selectivity theory [51]) by documenting the importance of close connections for older adults with regard to their mental health. 

### 4.1. Clinical Implications

Older adults are much more likely to suffer severe outcomes from COVID-19 infection [55], and higher depression and anxiety levels are associated with having a complex medical history [56]. In 2016, older adults in the U.S. averaged five visits per year to physician offices, which is significantly higher than the number of doctor visits for other adults, and most of those visits were for issues related to chronic conditions [57]. After the onset of the pandemic, in-person medical visits were largely replaced by telehealth visits [58,59], but in 2018 it was estimated that 38% of older adults would not be prepared to engage in video telehealth visits and about 20% would not be able to participate in telephone visits because of physiological or communication difficulties [60], which potentially limits opportunities to receive necessary medical care for a substantial number of older adults and may serve to increase anxiety and depression regarding their health. Given that telepsychological treatments for depression and anxiety have been found to be efficacious [61] and efficient [62,63], at-risk older adults could greatly benefit by utilizing telepsychology services. Interventions via telepsychology could offer treatment for mental health issues while simultaneously protecting those who are most physically vulnerable to COVID-19 by allowing them to receive treatment in the safety of their own homes. While vaccination efforts hold some promise of a return to a more normal way of life, it is still unclear how long it will be before it is safe for older adults to relax their social distancing precautions. For the foreseeable future, telepsychology may be the best option for providing psychological intervention for depression and anxiety in older adults. 

### 4.2. Limitations and Future Studies

A strength of this study was that it represented an international sample of older adults from multiple countries and regions. However, the sample was not an equal or populationally proportionate representation from each country, with a much larger number of respondents from the North America and Europe and Central Asia global regions than from the East Asia and Pacific and Sub-Saharan Africa Global Regions, so comparisons among global regions should be made with caution. The difference in representation and proportion of older adults may partially be due to the limited number of languages used for data collection and/or the snowball sampling method, which used mailing lists and social media (i.e., Facebook, Instagram, Twitter, and WhatsApp) to recruit participants. These limitations could also account for the relatively young older adult sample. Additionally, this was a cross-sectional study, and causal interpretations of relationships found should not be conclusively drawn. Future studies should endeavor to include older adults with a wider age range and a more equal distribution of participants across global regions. 

It would be enlightening to examine longitudinal data encompassing the entire period of the current pandemic to determine long-term effects on depression and anxiety in older adults, as research regarding the 2003 SARS outbreak in Canada found that prolonged quarantine time significantly contributed to psychological distress [64]. Likewise, a study from England examining effects of 2–4 months of lockdown in the current pandemic found that about 12% of cognitively healthy older adult participants reported increased depression symptoms and another 12% reported increased anxiety symptoms [6].

Older adults are disproportionately affected by COVID-19, and whether to protect older family members or to reduce their own risk, people are distancing themselves from older adults [65]. Exploration of the influences of ageism, racism, and classism on the mental health of older adults during the pandemic could be illuminating, as we already see emerging evidence of these biases and their effects [23] and racial/ethnic minority groups are more likely to have serious COVID-19-related illnesses [18]. 

## 5. Conclusions

The impacts of COVID-19 social distancing measures and the pandemic itself may be contributing to decreased mental wellbeing [8]. While older adults are generally better at emotion regulation than younger adults [66,67], thought to be due to prioritizing experiences that are personally meaningful and avoiding those which are stressful, the older adult advantage disappears when the omnipresence of the stressor is largely unavoidable [68], such as in the current COVID-19 pandemic. Although one study showed that older adults in the U.S. have maintained their emotion regulation advantage through the early stages of the pandemic by measuring the frequency and intensity of positive and negative emotions relative to younger adults [69], the current study has demonstrated that the changes brought about by COVID-19 have had serious deleterious effects on the mental health of older adults worldwide. Not only is it important for older adults to have access to their healthcare providers via telehealth technologies, using similar technological tools to maintain social networks may be crucial to the preservation and improvement of good mental health in older adults during the pandemic and beyond. It is therefore imperative that we increase older adult access to telehealth resources and improve available training in using associated technologies (and adaptations when necessary) in order to facilitate substantial improvements in the mental health of older adults. The current findings have direct implications for mental health services that may be delivered to older adults around the globe in order to help facilitate their healthy psychological adjustment during this challenging time.

## Figures and Tables

**Table 1:** Summary of Participant Demographic Characteristics.

Variable	*M* or *N*	*SD* or %
Age (years), *M, SD*	66.13	5.50
**Gender (*n* = 823), *n*, %**		
Man	210	25.5
Woman	610	74.1
Non-binary, transgender, or other	3	0.4
**Work status (*n* = 823), *n*, %**		
Active	339	41.2
Not active	484	58.8
**Marital status (*n* = 823), *n*, %**		
Partnered	521	63.3
Not partnered	302	36.7
**Dependents at home (*n* = 823), *n*, %**		
Children <18 years	25	3.0
No children	798	97.0
**Country income classification (*n* = 823), *n*, %**		
Lower-middle	1	0.1
Upper-middle	369	44.8
High	453	55.0
**Country region (*n* = 823), *n*, %**		
East Asia and Pacific	11	1.3
Europe and Central Asia	252	30.6
Latin America and the Caribbean	366	44.5
North America	173	21.0
Sub-Saharan Africa	21	2.6

**Table 2:** Correlation Matrix among Demographic Variables and Depression and Anxiety.

Variable	1	2	3	4	5	6	7
1. Anxiety							
2. Depression	0.737 ***						
3. Man vs. woman or non-binary/trans	−0.152 ***	−0.129 ***					
4. Age	−0.125 ***	−0.052	0.165 ***				
5. Country income classification	0.131 ***	0.247 ***	−0.097 **	0.132 ***			
6. Partnered vs. not partnered	−0.025	−0.088 *	0.185 ***	−0.019	−0.016		
7. Active vs. not active employment	0.060	−0.039	−0.014	−0.361 ***	−0.139 ***	0.002	
8. Dependent <18 years in home vs. not	−0.005	−0.023	0.075 *	−0.017	−0.067	0.047	0.010

Note. * = *p* < 0.05; ** = *p* < 0.01, *** = *p* < 0.001.

**Table 3:** Depression and Anxiety Multiple Regressions with Standardized β-weights Presented from Step 4.

						Model Significance			
		Depression		Anxiety		Depression		Anxiety	
Predictor Variable	%Yes/ Mean	β	*p*-Value	β	*p*-Value	*R* ^2^	*p*-Value	*R* ^2^	*p*-Value
**Step 1**						0.083	<0.001	0.052	<0.001
Man vs. woman or non–binary/trans	25.5%	−0.027	0.447	−0.050	0.161				
Age	66.13	−0.076	0.036	−0.101	0.006				
Country income classification	3.55	0.258	<0.001	0.184	<0.001				
Partnered vs. not partnered	63.3%	−0.062	0.072	0.002	0.948				
Employed vs. unemployed	41.2%	−0.064	0.109	−0.019	0.633				
Dependent <18 years old in home vs. not	3.0%	0.004	0.907	0.018	0.597				
**Step 2**						0.097	<0.001	0.068	<0.001
Currently have symptoms of this disease but have not been tested	1.1%	−0.013	0.713	0.010	0.773				
Tested and currently have this disease	0.4%	0.024	0.505	0.064	0.071				
Had symptoms of this disease but never tested	5.7%	0.033	0.364	0.011	0.753				
Tested positive for this disease but no longer have it	0.4%	−0.032	0.400	0.018	0.639				
Got medical treatment due to severe symptoms of this disease	0.7%	0.111	0.004	0.063	0.105				
Someone died of this disease while in our home	0.1%	0.013	0.704	−0.012	0.715				
Death of close friend or family member from this disease	1.8%	−0.034	0.320	−0.005	0.880				
**Step 3**						0.099	<0.001	0.070	<0.001
Quarantine level	3.68	0.047	0.182	0.046	0.191				
**Step 4**						0.174	<0.001	0.168	<0.001
Laid off from job or had to close own business	18.7%	−0.057	0.139	0.010	0.803				
Reduced work hours or furloughed	18.0%	0.069	0.061	0.021	0.566				
Had to continue to work even though in close contact with people who might be infected	7.8%	−0.010	0.797	0.026	0.481				
Provided direct care to people with the disease	3.6%	0.024	0.523	−0.003	0.928				
Increase in workload or work responsibilities	16.8%	−0.086	0.022	0.001	0.973				
Hard time doing job well because of needing to take care of people in the home	3.8%	0.041	0.237	0.035	0.314				
Hard time making the transition to working from home	13.1%	0.091	0.013	0.081	0.028				
Unable to get enough food or healthy food	6.4%	0.050	0.147	0.057	0.100				
Unable to pay important bills like rent or utilities	11.1%	0.058	0.108	0.066	0.068				
Had a child in home who could not go to school	3.6%	0.021	0.541	0.012	0.726				
Increase in verbal arguments or conflict with other adult(s) in home	9.6%	0.170	<0.001	0.216	<0.001				
Separated from family or close friends	74.5%	0.086	0.021	0.113	0.003				
Events/celebrations cancelled or restricted	80.6%	−0.022	0.548	−0.002	0.947				

**Table 4:** Depression and Anxiety Distribution by Country.

		Depression					Anxiety				
Country	*Total n*	Normal *n* (%)	Mild *n* (%)	Moderate *n* (%)	Severe *n* (%)	Extremely Severe *n* (%)	Normal *n* (%)	Mild *n* (%)	Moderate *n* (%)	Severe *n* (%)	Extremely Severe *n* (%)
Argentina	158	133 (84.2)	9 (5.7)	10 (6.3)	2 (1.3)	4 (2.5)	138 (87.3)	8 (5.1)	5 (3.2)	6 (3.8)	1 (0.6)
Australia	5	3 (60.0)	1 (20.0)	1 (20.0)	0	0	5 (100.0)	0	0	0	0
Austria	1	1 (100.0)	0	0	0	0	1 (100.0)	0	0	0	0
Belgium	28	13 (46.4)	3 (10.7)	6 (21.4)	1 (3.6)	5 (17.9)	22 (78.6)	2 (7.1)	1 (3.6)	2 (7.1)	1 (3.6)
Bolivia	1	1 (100.0)	0	0	0	0	1 (100.0)	0	0	0	0
Brazil	2	2 (100.0)	0	0	0	0	1 (50.0)	1 (50.0)	0	0	0
Canada	10	7 (70.0)	2 (20.0)	1 (10.0)	0	0	8 (80.0)	1 (10.0)	0	1 (10.0)	0
Chile	23	18 (78.3)	3 (13.0)	1 (4.3)	0	1 (4.3)	20 (87.0)	2 (8.7)	1 (4.3)	0	0
Colombia	53	44 (83.0)	3 (5.7)	5 (9.4)	0	1 (1.9)	50 (94.3)	0	2 (3.8)	0	1 (1.9)
Costa Rica	2	2 (100.0)	0	0	0	0	2 (100.0)	0	0	0	0
Croatia	1	1 (100.0)	0	0	0	0	1 (100.0)	0	0	0	0
Cyprus	1	0	0	1 (100.0)	0	0	1 (100.0)	0	0	0	0
Dominican Republic	3	3 (100.0)	0	0	0	0	3 (100.0)	0	0	0	0
Ecuador	8	5 (62.5)	0	1 (12.5)	2 (25.0)	0	6 (75.0)	1 (12.5)	0	1 (12.5)	0
France	53	16 (30.2)	11 (20.8)	7 (13.2)	7 (13.2)	12 (22.6)	32 (60.4)	6 (11.3)	10 (18.9)	4 (7.5)	1 (1.9)
Germany	31	19 (61.3)	6 (19.4)	4 (12.9)	2 (6.5)	0	30 (96.8)	1 (3.2)	0	0	0
Guatemala	2	2 (100.0)	0	0	0	0	2 (100.0)	0	0	0	0
Hungary	1	0	1 (100.0)	0	0	0	1 (100.0)	0	0	0	0
Italy	19	7 (36.8)	4 (21.1)	4 (21.1)	2 (10.5)	2 (10.5)	15 (78.9)	1 (5.3)	2 (10.5)	0	1 (5.3)
Japan	4	4 (100.0)	0	0	0	0	4 (100.0)	0	0	0	0
Mexico	67	57 (85.1)	4 (6.0)	4 (6.0)	2 (3.0)	0	61 (91.0)	4 (6.0)	1 (1.5)	1 (1.5)	0
Netherlands	1	1 (100.0)	0	0	0	0	1 (100.0)	0	0	0	0
New Zealand	2	1 (50.0)	1 (50.0)	0	0	0	2 (100.0)	0	0	0	0
Panama	6	4 (66.7)	1 (16.7)	1 (16.7)	0	0	4 (66.7)	1 (16.7)	1 (16.7)	0	0
Paraguay	4	4 (100.0)	0	0	0	0	4 (100.0)	0	0	0	0
Peru	21	17 (81.0)	2 (9.5)	1 (4.8)	1 (4.8)	0	18 (85.7)	2 (9.5)	1 (4.8)	0	0
South Africa	21	15 (71.4)	3 (14.3)	2 (9.5)	1 (4.8)	0	18 (85.7)	1 (4.8)	2 (9.5)	0	0
Spain	46	32 (69.6)	5 (10.9)	5 (10.9)	4 (8.7)	0	38 (82.6)	3 (6.5)	2 (4.3)	3 (6.5)	0
Turkey	20	6 (30.0)	5 (25.0)	7 (35.0)	0	2 (10.0)	16 (80.0)	0	3 (15.0)	0	1
United Kingdom	50	20 (40.0)	7 (14.0)	12 (24.0)	5 (10.0)	6 (12.0)	36 (72.0)	1 (2.0)	10 (20.0)	1 (2.0)	2 (4.0)
United States	163	108 (66.3)	20 (12.3)	19 (11.7)	9 (5.5)	7 (4.3)	136 (83.4)	3 (1.8)	15 (9.2)	7 (4.3)	2 (1.2)
Uruguay	8	4 (50.0)	3 (37.5)	1 (12.5)	0	0	8 (100.0)	0	0	0	0
Venezuela	8	6 (75)	1 (12.5)	1 (12.5)	0	0	7 (87.5)	0	1 (12.5)	0	0

## Data Availability

The data presented in this study are available on request from the corresponding author. The data are not publicly available due to privacy issues.
